# Data-driven questionnaire-based cluster analysis of asthma in Swedish adults

**DOI:** 10.1038/s41533-020-0168-0

**Published:** 2020-04-06

**Authors:** Marta A. Kisiel, Xingwu Zhou, Josefin Sundh, Björn Ställberg, Karin Lisspers, Andrei Malinovschi, Hanna Sandelowsky, Scott Montgomery, Anna Nager, Christer Janson

**Affiliations:** 1 Department of Medical Sciences: Environmental and Occupational Medicine, Uppsala University, Uppsala, Sweden; 2 Department of Medical Sciences: Respiratory, Allergy and Sleep Research, Uppsala University, Uppsala, Sweden; 3 Department of Medical Sciences: Clinical Physiology, Uppsala University, Uppsala, Sweden; 4 Department of Public Health Sciences, Karolinska Institute, Stockholm, Sweden; 5 Department of Respiratory Medicine, Faculty of Medicine and Health, Örebro University, Örebro, Sweden; 6 Department of Public Health and Caring Sciences, Family Medicine and Preventive Medicine, Uppsala University, Uppsala, Sweden; 7 NVS, Section for Family Medicine and Primary Care, Karolinska Institute, Stockholm, Sweden; 8 Clinical Epidemiology and Biostatistics, School of Medical Sciences, Örebro University, Örebro, Sweden; 9 Clinical Epidemiology Division, Department of Medicine, Karolinska Institute, Stockholm, Sweden; 10 Department of Epidemiology and Public Health, University College London, London, UK

**Keywords:** Health care, Respiratory tract diseases

## Abstract

The aim of this study was to identify asthma phenotypes through cluster analysis. Cluster analysis was performed using self-reported characteristics from a cohort of 1291 Swedish asthma patients. Disease burden was measured using the Asthma Control Test (ACT), the mini Asthma Quality of Life Questionnaire (mini-AQLQ), exacerbation frequency and asthma severity. Validation was performed in 748 individuals from the same geographical region. Three clusters; early onset predominantly female, adult onset predominantly female and adult onset predominantly male, were identified. Early onset predominantly female asthma had a higher burden of disease, the highest exacerbation frequency and use of inhaled corticosteroids. Adult onset predominantly male asthma had the highest mean score of ACT and mini-AQLQ, the lowest exacerbation frequency and higher proportion of subjects with mild asthma. These clusters, based on information from clinical questionnaire data, might be useful in primary care settings where the access to spirometry and biomarkers is limited.

## Introduction

Despite progress in asthma treatment, the number of patients with poorly controlled disease remains high^1^. One reason for this is the heterogeneous character of the disease, involving complex pathophysiological processes^2^.

The heterogeneity in asthma is manifested by varying risk of exacerbation and the inconsistent response to therapy^3^. In order to optimize management for patients with different disease severity, asthma has been classified into groups (phenotypes). Phenotype is defined as an observable characteristic of a subject resulting from the interaction between the genotype and environmental factors^4^. Because asthma stratification is based on complex not linear combination of symptoms, lung function and treatment protocols, unbiased cluster analysis has been found to be a useful method for identification of asthma clusters. The majority of previous research has been conducted using multiple measurements such as sputum, serum and bronchoalveolar lavage fluid cell counts and biomarkers, exhaled nitric oxide (FENO), pulmonary function tests and genetic data^5,6^. The majority of those phenotypes have been studied in patients severe asthma and have not been validated in an independent cohort. Meanwhile, the majority of patients with asthma are detected, treated and followed-up in primary care where there usually is no access to complex data^3,7^.

Despite several previously identified phenotypes of severe asthma, the clinical perspective on asthma outcome has not been sufficiently optimized^8^. Furthermore, the correlation between severity of asthma and molecular findings is low^9^. Only a few attempts have been made to identify clinical phenotypes in mild-to-moderate asthma that might be applicable in asthma patients in primary care^10–13^.

In the current study, we aimed to characterize asthma phenotypes by using a limited set of variables obtained from a patient’s questionnaire that can be easily collected and used in routine primary care practice. We applied unsupervised cluster analysis that enabled us to stratify data set without any previously defined hypothesis. The results reproducibility were further studies in one independent population of asthma patients.

## Results

### Study population

The baseline characteristics were quite similar in both cohorts as shown in Table 1. We did not detect any significant difference for most of the variables, except that the mean age of participants was slightly higher and reported allergy against pollen and pets slightly lower in the validation cohort than in the discovery cohort. Approximately two-thirds of all patients with asthma were under the care of a primary care physician. The quality of the data was good, and the proportion of the missing values for the variables quite low (Table 1). Imputation was not used for missing values.

**Table 1:** Baseline characteristics of the asthma discovery and validation cohort.

Patient’s characteristics	Discovery cohort (*N* = 1291)		Validation cohort (*N* = 748)		*p* values
	Descriptive statistics	Missing values	Descriptive statistics	Missing values	
Sex; % female	61.4	0	60.0	0	0.64
Age in years; mean (±SD)	54.3 (15.5)	0	57.8 (14.4)	0	<0.01
BMI; mean (±SD)	27.5 (5.7)	5.1	27.4 (4.9)	4.0	0.51
Age of onset, %					
≤15 years	36.7	3.3	33.2	0.05	0.45
16–45 years	38.7		40.3		
≥46 years	24.6		26.5		
Allergy against pollen or pets, %	37.5	0.3	30.1	0.4	0.01
Rhinitis, %	70.7	0.5	66.0	0.1	0.12
Diabetes, %	8.1	0	9.6	0	0.45
Cardiovascular disease, %	30.9	0	32.5	0	0.63
Depression and anxiety, %	14.9	0	12.6	0	0.45
Sleep apnea, %	8.8	0	7.0	0	0.45
Gastroesophageal reflux, %	17.4	0	18.7	0.4	0.63
Sinusitis, %	21.0	0	18.6	2.4	0.45
Night awakening last week, %					
Never	69.6	1.8	69.7	2.0	0.97
Once	12.5		12.1		
More than one	17.9		18.1		
Smoking, %					
Never	56.7	1.2	55.4	1.5	0.69
Ex	32.6		34.6		
Current	10.7		10.0		
Education level, %					
Compulsory school	25.0	1.3	35.6	2.0	<0.01
Secondary school	38.4		34.4		
High education	36.6		30.0		
Physical activity, %					
Daily	9.0	3.2	6.7	2.1	0.45
Few times a week	64.8		65.3		
Once a month	5.6		6.4		
Less	20.6		21.6		

Age and BMI were tested by ANOVA, other variables were tested by the chi-square test. The *p* values were adjusted according to the Benjamini & Hochberg method and considered significant when <0.05.

### Cluster analysis in discovery cohort

The cluster analysis of the discovery cohort resulted in identification of three clusters (phenotypes), as three clusters gave the highest silhouette distance. The majority of the parameters were significantly different between the phenotypes (Supplementary Figs. 1 and 2, Table 2).

**Table 2:** Summary of the patient characteristics of three phenotypes obtained after the cluster analysis of the discovery cohort.

Discovery cohort				
Patient’s characteristics	Early onset predominantly female (*N* = 526)	Adult onset predominantly female (*N* = 451)	Adult onset predominantly male (*N* = 314)	*p* values
Sex; % female	70.7	70.7	32.5	<0.001
Age in years; mean (±SD)	46.5 (15.1)	60.5 (12.0)	58.6 (15.1)	<0.001
BMI; mean (±SD)	26.9 (5.8)	28.7 (6.0)	27.3 (4.9)	<0.001
Age of onset, %				
≤15 years	58.7	18.9	24.8	<0.001
16–45 years	33.1	47.1	35.9.	
≥46 years	8.1	34.0	39.3	
Allergy against pollen or pets, %	73.1	15.6	8.9	<0.001
Rhinitis, %	90.2	81.0	23.3	<0.001
Diabetes, %	2.9	15.5	6.4	<0.001
Cardiovascular disease, %	9.5	64.3	18.8	<0.001
Depression and anxiety, %	17.3	16.4	8.6	0.002
Sleep apnea, %	5.9	13.3	7.3	<0.001
Gastroesophageal reflux, %	18.7	19.5	12.1	0.019
Sinusitis, %	34.1	13.3	10.1	<0.001
Night awakening last week, %				
Never	61.8	71.1	80.7	<0.001
Once	18.2	10.5	5.6	
More than one	20.0	18.4	13.7	
Smoking, %				
Never	68.5	35.7	67.0	<0.001
Ex	20.4	50.8	27.2	
Current	11.2	13.5	5.8	
Education level, %				
Compulsory school	15.8	32.7	29.4	<0.001
Secondary school	41.9	34.8	37.7	
High education	42.3	32.5	32.9	
Physical activity, %				
Daily	9.4	9.8	7.5	0.496
Few times a week	66.8	61.2	66.6	
Once a month	5.1	5.8	6.2	
Less	15.1	23.3	19.8	

Age and BMI were tested by ANOVA, other variables were tested by the chi-square test. The *p* values were adjusted according to the Benjamini & Hochberg method and considered significant when <0.05.

Phenotype 1 defined as early onset predominantly female asthma was characterized by younger age, female predominance, early onset disease, high prevalence of night awakenings due to asthma symptoms, reported allergy against pollen and pets, rhinitis and sinusitis, slightly lower BMI and low prevalence of diabetes, cardiovascular disease and sleep apnea. This group had the highest percent of subjects with a high education.

Phenotype 2 defined as adult onset predominantly female asthma included predominantly women of higher age and most had an adult onset asthma. The prevalence of allergy against pollen and pets was low whereas the prevalence of rhinitis, cardiovascular disease, diabetes and sleep apnea was high. This phenotype also had the highest rate of current and ex-smoker as well as the highest rate of subjects with only compulsory education and the highest proportion of participants that exercised less than once a month.

Phenotype 3 defined as adult onset predominantly male asthma included predominantly men with adult onset asthma and a low prevalence of allergy and rhinitis. The rate of sinusitis, gastroesophageal reflux disorder (GERD), depression/anxiety, night awaking due to asthma was the lowest in this group. This group included the highest proportion of patients that reported no current asthma, defined as no current asthma medication and asthma symptoms.

### Comparison of discovery and validation cohort

We used the second independent asthma cohort, the validation cohort, to repeat the cluster analysis (Table 3). In this population, the optimal number of clusters was also three (Supplementary Figs. 1 and 2). The majority of characteristics of the cluster-generated phenotypes corresponded to those in the discovery cohort with a few exceptions. The mean age of subjects with early onset predominantly female asthma in the validation population was higher in comparison to corresponding group in the discovery cohort. There were also some differences in the prevalence of cardiovascular disease, diabetes, sleep apnea and smoking habits. More specifically, the incidence rate of all cardiovascular disease, diabetes and sleep apnea was the highest in adult onset predominantly female asthma and the lowest in adult onset predominantly male asthma in the discovery cohort. In contrast, in the validation cohort, diabetes and cardiovascular disease was the highest in adult onset predominantly male asthma and the lowest in the early onset predominantly female phenotype while sleep apnea was quite similar across the groups. The distribution of smoking habits varied in both populations. There were more never smokers in both phenotypes with adult onset asthma in the discovery cohort than in the validation cohort. The rate of ex-smokers was higher in the adult onset predominantly female asthma but lower in adult onset predominantly male asthma in the discovery cohort than in the validation cohort.

**Table 3:** Summary of the patient characteristics of three phenotypes obtained after the cluster analysis of the validation cohort.

Validation cohort				
Patient’s characteristics	Early onset predominantly female (*N* = 234)	Adult onset predominantly female (*N* = 298)	Adult onset predominantly male (*N* = 216)	*p* values
Sex; % female	71.4	69.1	35.2	<0.001
Age in years; mean (±SD)	54.4 (14.6)	57.6 (13.9)	61.8 (13.7)	<0.001
BMI; mean (±SD)	27.1 (5.0)	27.8 (5.1)	27.2 (4.6)	0.239
Age of onset, %				
≤15 years	45.4	31.2	22.1	<0.001
16–45 years	41.4	43.4	34.7	
≥46 years	13.2	25.3	43.2	
Allergy against pollen or pets, %	87.6	0.7	8.4	<0.001
Rhinitis, %	93.6	78.2	19.4	<0.001
Diabetes, %	8.5	9.7	10.6	0.814
Cardiovascular disease, %	27.8	34.2	35.2	0.233
Depression and anxiety, %	17.9	12.1	7.4	0.005
Sleep apnea, %	7.3	7.4	6.0	0.814
Gastroesophageal reflux, %	25.0	18.5	12.0	0.004
Sinusitis, %	28.9	16.7	10.0	<0.001
Night awakening last week, %				
Never	64.8	68.4	76.9	0.082
Once	12.6	13.1	10.4	
More than one	22.6	18.6	12.7	
Smoking, %				
Never	68.4	48.6	50.5	<0.001
Ex	24.7	38.4	40.2	
Current	6.9	13.0	9.3	
Education level, %				
Compulsory school	26.2	37.1	43.8	0.003
Secondary school	37.6	34.7	30.5	
High education	36.2	28.2	25.7	
Physical activity, %				
Daily	7.4	5.1	8.1	0.814
Few times a week	66.5	66.8	61.9	
Once a month	6.1	6.8	6.2	
Less	20.0	21.2	23.8	

Age and BMI were tested by ANOVA, other variables were tested by the chi-square test. The *p* values were adjusted according to the Benjamini & Hochberg method and considered significant when <0.05.

### Disease burden

**Fig. 1:**
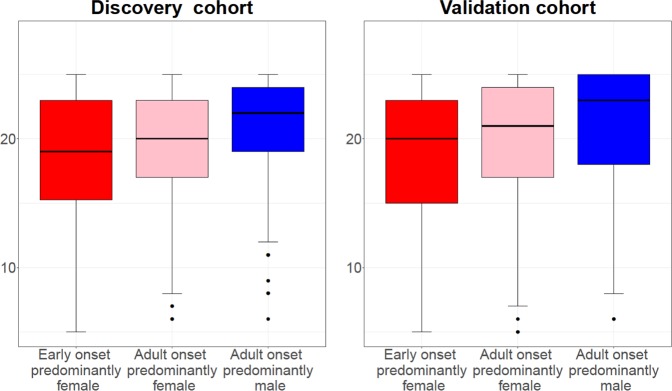
The median of ACT score in three phenotypes in both cohorts. The hinge denotes the 50% percentiles of the observed data for each phenotype. The whisker (upper or lower) extends from the hinge to maximum 1.5 times of the interquartile range, or IQR. The outliers are those beyond the end of the whiskers and are plotted individually.

**Fig. 2:**
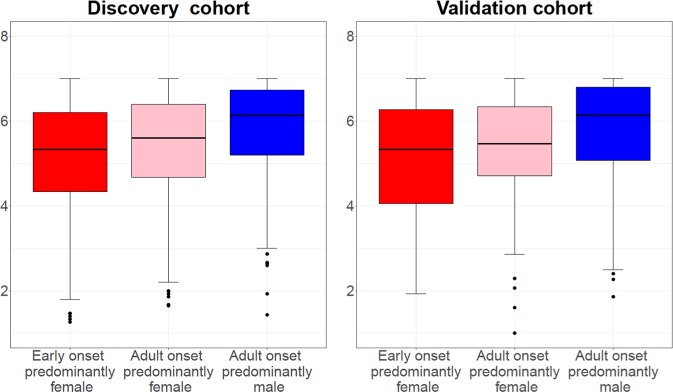
The median of mini-AQLQ score in three phenotypes in both cohorts. The hinge denotes the 50% percentiles of the observed data for each phenotype. The whisker (upper or lower) extends from the hinge to maximum 1.5 times of the interquartile range, or IQR. The outliers are those beyond the end of the whiskers and are plotted individually.

**Fig. 3:**
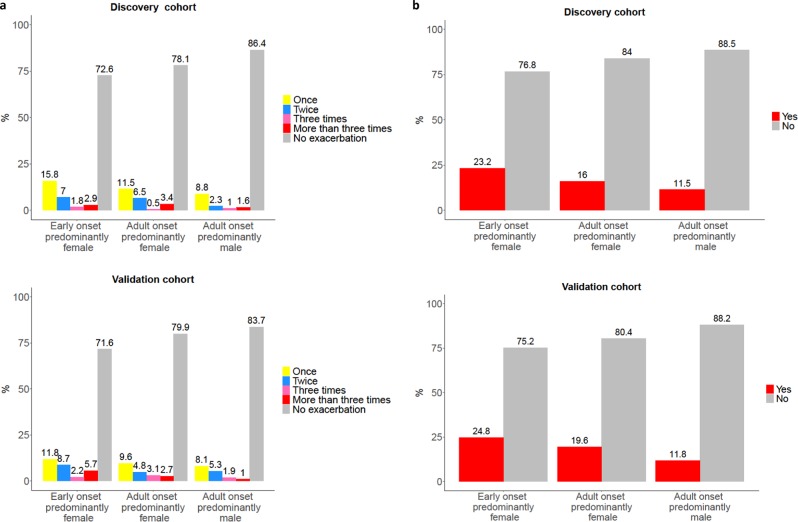
The exacerbation rate in three phenotypes in both cohorts. **a** The mean rate of exacerbation (%) defined as the number of asthma-related emergency visits due to asthma in the last 12 months. **b** The mean rate of exacerbation (%) defined as the usage of oral corticosteroids as prednisolone and betamethasone.

**Fig. 4:**
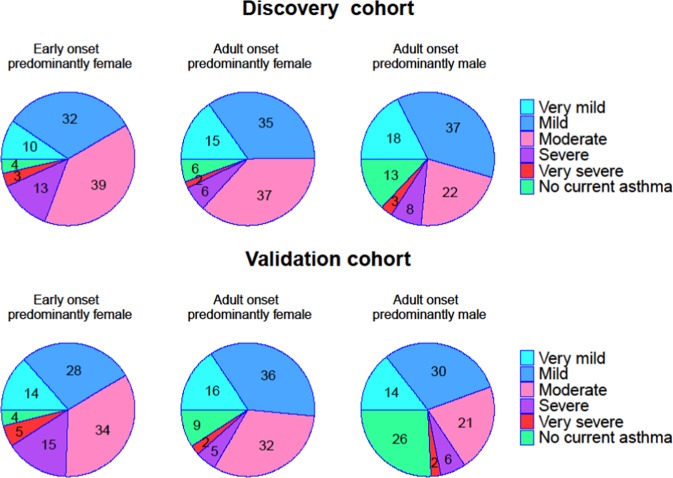
The patients self-report of asthma severity divided into very mild, mild, moderate, very severe and no current asthma. The proportion of individuals in each of three phenotypes is presented.

The results concerning mini-AQLQ, ACT, exacerbation frequency, defined as either the number of emergency visits due to asthma in the last 12 months or usage of OCS during the last 6 months, self-reported asthma severity and treatment steps, had a similar pattern in both populations (Supplementary Table 2, Figs. 1–4). Early onset predominantly female asthma had lowest mean score of mini-AQLQ and the highest rate of exacerbations, while adult onset predominantly male phenotype had the highest mean score of ACT and mini-AQLQ and the lowest rate of exacerbations. A higher proportion of patients in early onset predominantly female asthma used inhaled corticosteroids (ICS) compared to the other two phenotypes. Adult onset predominantly male asthma had the higher rate of patients without ICS treatment (Table 4). In this group the proportion of patients reporting very mild or mild asthma was the highest. Additionally, this phenotype also had the highest proportion of patients reporting no current asthma (Fig. 4).

**Table 4:** The asthma treatment used by the patients in both cohorts and each of three phenotypes of the discovery and validation cohort.

Medication %	Discovery cohort			Validation cohort		
	Early onset predominantly female	Adult onset predominantly female	Adult onset predominantly male	Early onset predominantly female	Adult onset predominantly female	Adult onset predominantly male
No ICS	38.0	45.7	52.8	40.5	46.4	53.6
Only ICS	18.3	16.8	14.6	11.0	16.2	15.3
ICS and (LABA and/or LTRA)	43.7	37.5	32.6	48.5	37.5	31.1

*ICS* inhaled corticosteroids, *LABA* long acting beta-2-agonists, *LTRA* leukotrienereceptor antagonists.

## Discussion

In this study we performed a cluster analysis of two independent population of asthma patients. By using the patients-reported data of the discovery cohort (*n* = 1291), we developed the cluster model and identified three phenotypes with distinct demographic and clinical characteristics. Then, we repeated the cluster analysis in a second independent population, the validation cohort (*n* = 748) where we were able to replicate the division into three similar groups. The disease burden was measured by validated instruments such as ACT, mini-AQLQ as well as by self-reported exacerbation, patients-reported severity and treatment.

Our main finding was that both cohorts consisted of three corresponding phenotypes of asthma patients that had different levels of asthma control and asthma-related quality of life. In line with the previous studies we found that sex and age of asthma onset were key factors across the three identified groups^3,5^. Therefore, we named the phenotypes as early onset predominantly female, adult onset predominantly female, and adult onset predominantly male asthma. Adult onset predominantly male asthma with a low rate of atopy had well-controlled asthma and the highest rate of patients without ICS treatment. In this phenotype we found the highest proportion of patients that reported no current asthma as they had no ongoing asthma medication or symptoms. The poorest asthma control and the highest use of ICS was found in early onset predominantly female, also characterized by a high rate of atopy. This finding was similar to Haldar et al.^12^, who performed the first cluster analysis on primary care data. They reported that patients with early onset atopic asthma had the worst outcomes. In contrast, in the study by Khusial et al. the phenotype early atopic had the most favorable outcomes of AQLQ, asthma control and used the lowest dosage of ICS. However, in Khusial’s study, the exacerbation phenotype was distinguished as a separate group of patients. Interestingly, the early atopic included an approximately equal number of men and women while exacerbators consist of 75% females^10^.

In the present study early onset predominantly female cluster had the lowest asthma control, had the highest prevalence of GERD, sinusitis, depression and anxiety. This is in accordance with previous studies that found that GERD and sinusitis were positively correlated to higher severity and poor control of the disease^5,11,14^ and similar to earlier reports that depression and anxiety were risk factors for severe asthma^15^. There was some difference between the cohorts and corresponding phenotypes regarding the prevalence of diabetes and cardiovascular disease. The higher age of patients in the validation population, particularly in early onset predominantly female phenotype, might be the reason for the higher prevalence of cardiometabolic disease in this population. To our knowledge our study is the first cluster analysis evaluating associations of cardiometabolic conditions in relation to phenotypes of asthma in primary care. The highest rate of current smokers was found in adult onset predominantly female asthma and the highest rate of never smokers in early onset predominantly female asthma. Previous studies showed that smoking was less common in people with higher educational level^16^ and the highest rate of patients with university education was found in early onset predominantly female asthma. Notably, we did not find any differences between reported physical activities between the phenotypes.

A few previous papers have reported results from cluster analysis in two independent populations. One example is Siroux et al.^9^ that used a French case-control and family-based study (EGEA2) and the European Community Respiratory Health survey II and identified four asthma phenotypes, mainly based on either atopy or the age of onset. A few studies have attempted to replicate the findings of earlier studies. Savenije et al.^17^ applied the same phenotypes in the Dutch children (*n* = 3789) as previously identified by an unsupervised statistical approach in the UK in the Avon Longitudinal Study of Parents and Children^18^. As a result, five of six phenotypes identified in ALSPAC was successfully replicated. However, 63% of the UK primary care patients in a 2019 study of Nissen et al.^19^ did not fit in any of the phenotypes that were described in a mix of UK primary and secondary care patients in a study of Haldar et al.^12^ 10 years earlier.

We propose to use this cluster classification to identify patients at risk at primary health-care centers. In order to facilitate asthma patient classification, patients with newly diagnosed asthma could be asked to answer some questions by a mobile app or online questionnaire. Different asthma phenotypes may need different levels of management. Early onset predominantly female asthma had the highest disease burden. Because of this perhaps primary care attention should direct more attention on early onset predominantly female. These patients may require frequent visits and tight contact with asthma nurses. In contrast to this, adult onset predominantly male asthma has the most favorable results. These patients might not need health-care visits and could instead be asked to complete the questionnaire every third month. This would facilitate optimal use of the physicians and nurse resources.

The main strength of our study is the use of two randomly selected large cohorts with asthma, both collected at the health-care centers (mainly in the primary care) close in time. Therefore, the number of patients per phenotype was quite large. Our study was completely based on the questionnaire. Another strength was that our study was based on self-reported data from standardized protocols and validated questionnaires, which can give a more nuanced picture than register data. Previously only Ortega et al.^11^ had based the cluster analysis with asthma patients only on questionnaire data. However, that study used the hypothesis-driven (supervised) cluster analysis that had clearly defined aim (to determine the phenotype at the highest risk of exacerbation)^11^. Instead, we performed Partitioning Around Medoids (PAM), an unsupervised (data-driven) cluster analysis without previously defined hypothesis. The PAM method is robust and suitable when having both numerical and categorical data, while most other traditional methods such as k-means or hierarchical clustering methods were limited to numerical data, and latent class methods work are limited to categorical data. We based our study on standardized protocols and validated outcome tools, such as mini-AQLQ and ACT, that made our results stronger. The exacerbation rate was one of the outcome variables that was used to control if the groups were related to the clinical outcome. It would, however, also be interesting to make a model where exacerbation rate was included in the cluster analysis as it is a variable that is clinically easy to collect. One problem with the use of self-reported data is that patients may differ in understanding, defining, and remembering^20^. Another limitation was that both the cohorts were collected in the same region of Sweden and they may not represent the whole Swedish population. Furthermore, we did not have access to pulmonary function measures that some researchers consider as one of the most important discriminant variables in cluster analysis of asthma^5,21,22^. On the other hand, spirometric measurements and other laboratory variables may not be available in all primary care centers^3,23^.

In conclusion, we were able to detect three distinct patient phenotypes of asthma, similarly in two independent populations, by using cluster analysis. Moreover, the clustering was based on patient-reported data rather than biomarkers, which increases the feasibility and clinical use of the method, particularly in primary care.

## Methods

### Data collection

This study is based on two independent Swedish asthma cohorts from the PRAXIS study collected in 2012 and 2015. Characteristics of the two patient populations were reported in more detail in previous publication^24^. Briefly, both cohorts were identified for research purpose in seven country councils in central Sweden. Eight randomly selected primary health-care centers, one randomly selected department of internal medicine and one respiratory medicine department were included in each council. Randomly selected adult patients, with a doctor’s diagnosis of asthma (ICD-10 code J45) in the medical records, were sent a questionnaire. The first, discovery cohort comprised patients that were contacted for the first time in 2015. The response rate was 46% (*n* = 1291) where 915 subjects were from primary care and 376 were from hospitals^24^. The second, validation cohort was used to confirm the results of the analyses in the discovery cohort. The validation cohort answered in 2005 a brief postal questionnaire and in 2012 a more extensive patient questionnaire that was identical to the one used in the validation cohort^25^. The response rate was 62% (*n* = 750). Two patients were excluded as they were already in the first cohort that resulted in 748 subjects.

The study protocols were approved by the regional ethics review committee in Uppsala (DNr 2011/318). All participants gave written informed consent.

### Questionnaire

The PRAXIS questionnaire collected information on demographic, self-reported asthma characteristics and other relevant information^24^. For this study we used selected items from this questionnaire (Supplementary Table 1). The selection of variables was based on previous cluster analysis in primary care^10–12^. We included age, sex, body mass index (BMI in kg/m^2^) and smoking status (never smoker, ex-smoker and current smoker included current daily smoker and occasional smoker). In order to determine disease severity, we added age of onset (≤15; 17–45; ≥46 years), night awakening due to asthma symptoms (cough, wheeze and/or dyspnea), reported allergy against pollen and pets and rhinitis. Additionally, we included co-morbidity with cardiovascular conditions (high blood pressure, heart disease and stroke), diabetes, depression and/or anxiety, sleep apnea, GERD and sinusitis. Information on the educational status and the physical activity was also collected.

### Outcome variables

The following variables were used to identify differences in the different cluster-generated groups: mini Asthma Quality of Life Questionnaire (mini-AQLQ)^26^, Asthma Control Test (ACT)^27^, exacerbation rate defined as asthma-related emergency visits in primary and/or secondary care in the last 12 months or defined as the use of per oral corticosteroids (OCS: prednisolone or betamethasone) due to asthma symptoms during the last 6 months, patients-reported severity of asthma (no current asthma/very mild/mild/moderate/severe) and maintenance treatment steps grouped such as (a) no inhaled corticosteroids (ICS); (b) ICS alone; (c) ICS and long acting beta-2 agonist and/or leukotriene antagonist (Supplementary Table 1).

### Data-driven cluster analysis, statistic

Data-driven cluster analysis was performed in the discovery cohort. The cluster analysis was then repeated in the validation cohort. Fourteen variables including continuous variables (age, BMI) and categorical variables (sex, age of onset, allergy against pollen or pets, rhinitis, diabetes, cardiovascular disease, depression and anxiety, sleep apnea, GERD, sinusitis, night awaking last week, smoking) were selected for clustering analysis. All the variables are listed in Table 1. The PAM analysis was selected to conduct the analysis mostly because we have both continuous variables and categorical variables, and PAM is well-known for robustness and suitable for arbitrary distance^28^, while other clustering methods are limited to either numerical data or categorical data.

The cluster analysis includes the following steps:

1. The Gower distance was used to construct the dissimilarity matrix, since there was a mixture of numerical variables and categorical variables^29^.
2. The number of clustering was selected according to the silhouette distance. To get the optimal number of clusters, the number of clusters from 2 to 10 was tried, and the one that generated the largest silhouette width was selected (Supplementary Fig. 1).
3. The PAM analysis was conducted by the pam function in the cluster package in R version 3.5.3)^30^.

### Statistical analysis

The categorical variables were calculated as percent within one cohort or one group. The numerical variables, including age, BMI, ACT and mini-AQLQ, were presented as mean and SD. The outcome variables as mini-AQLQ and ACT were shown as median and interquartile range (IQR). To study if there are differences among the clusters, ANOVA analysis was used for the numerical variables (age, BMI) and chi-square test was applied for the categorical variables (other 12 categorical variables). The *p* values were corrected for multiple comparisons by the Benjamini & Hochberg method^31^. The Spearman rank correlation was calculated to detect the association between the self-reported severity and the ACT score/exacerbation history.

### Reporting summary

Further information on experimental design is available in the Nature Research Reporting Summary linked to this article.

## Supplementary information

Reporting summary

Supplementary Info

## Data Availability

All data that support the findings of this study have been deposited in the PRAXIS database (https://www.researchweb.org/is/fourol/project/157771) and it is available on request from the authors.
