# Supplementary material for: Data-driven questionnaire-based cluster analysis of asthma in Swedish adults
Source: NPJ Prim Care Respir Med. 2020 Apr 6;30:14. doi: 10.1038/s41533-020-0168-0 (PMC7136224; doi:10.1038/s41533-020-0168-0)

Supplementary material:

Supplementary Table 1. The items from the PRAXIS questionnaire used in the study. ICS – inhaled corticosteroids; LABA- long acting beta-2 agonists; LTRA - leukotriene antagonist. ACT-asthma Control Test; mini-AQLQ – mini Asthma Quality of Life Questionnaire.

	ITEMS	ANSWERS
**Variables into the cluster model**		
1	How old are you?	....years
2	Sex	Male….Female….
3	Length and weight	…cm ….kg
4	Do you smoke?	Never/Ex/Current (daily or occasional)
5	Age of asthma onset	<=15 years/16-45 years/>=46 years
6	Do you have allergy against pets or pollen?	Yes often/Sometimes only or No
7	Have you had rhinitis in the last 12 months?	Yes/No
8	Do you have gastroesophageal reflux in the night (at least once a week)?	Yes/No
9	Have you had diabetes in the last 12 months?	Yes/No
10	Have you had cardiovascular disease (heart disease, stroke and hypertension) in the last 12 months?	Yes/No
11	Have you had sleep apnea in the last 12 months?	Yes/No
12	Have you had depression and/or anxiety in the last 12 months?	Yes/No
13	Have you had sinusitis in the last 6 months?	Yes/No
14	Have you woken up in the night due to asthma symptoms such as cough, wheeze and/or dyspnea (during the last week)?	No/Once/ Several times
**Not included into the cluster model**		
1	How often do you exercise (until you get out of breath or/and sweat)?	Daily/few times a week/Once a week/Once a month/Less
2	What is your highest education level?	Compulsory school (up to 15 years old)<br>Secondary school (up to 18 years old)<br>High education (at least 3 years old beyond secondary level)
**Outcome variables**		
1	ACT	Reference[27]
2	Mini-AQLQ	Reference[26]
3a	Have you visited emergency due to asthma related symptoms during the last 12 months?	Never/Once/Twice/Three or more times
3b	Have you used oral corticosteroids (prednisolone or betamethasone) because of asthma symptoms during the last 6 months?	Yes/No
4	Patients reported asthma severity	No current asthma/Very mild/mild/moderate/severe
5	Treatment steps	(a) No ICS<br>(b) Only ICS<br>(c) ICS+LABA and/or LTRA

Supplementary Table 2. The mean score (±SD) of ACT and mini-AQLQ in three phenotypes in both cohorts.

Cohort		Early onset predominantly female	Adult onset predominantly female	Adult onset predominantly male
**Discovery**	**ACT**	18.7 (4.6)	19.4 (4.7)	21.0 (4.3)
	**Mini-AQLQ**	5.1 (1.3)	5.4 (1.2)	5.8 (1.1)
**Validation**	**ACT**	18.8 (5.1)	19.9 (4.6)	20.9 (4.5)
	**Mini-AQLQ**	5.1 (1.4)	5.4 (1.1)	5.8 (1.2)

Supplementary Table 3. The Spearman rank correlation between the self-reported severity and the ACT-score/exacerbation history

**Cohort**		**Correlation coefficients**	**P-values**
**Discovery**	**Self--ACT**	-0.61	p<0.001
	**Self--exacerbation**	0.35	p<0.001
**Validation**	**Self-ACT**	-0.67	p<0.001
	**Self--exacerbation**	0.41	p<0.001

Supplementary Figure 1. The silhouette distance analysis suggests the optimal number of clusters (phenotypes) as three.

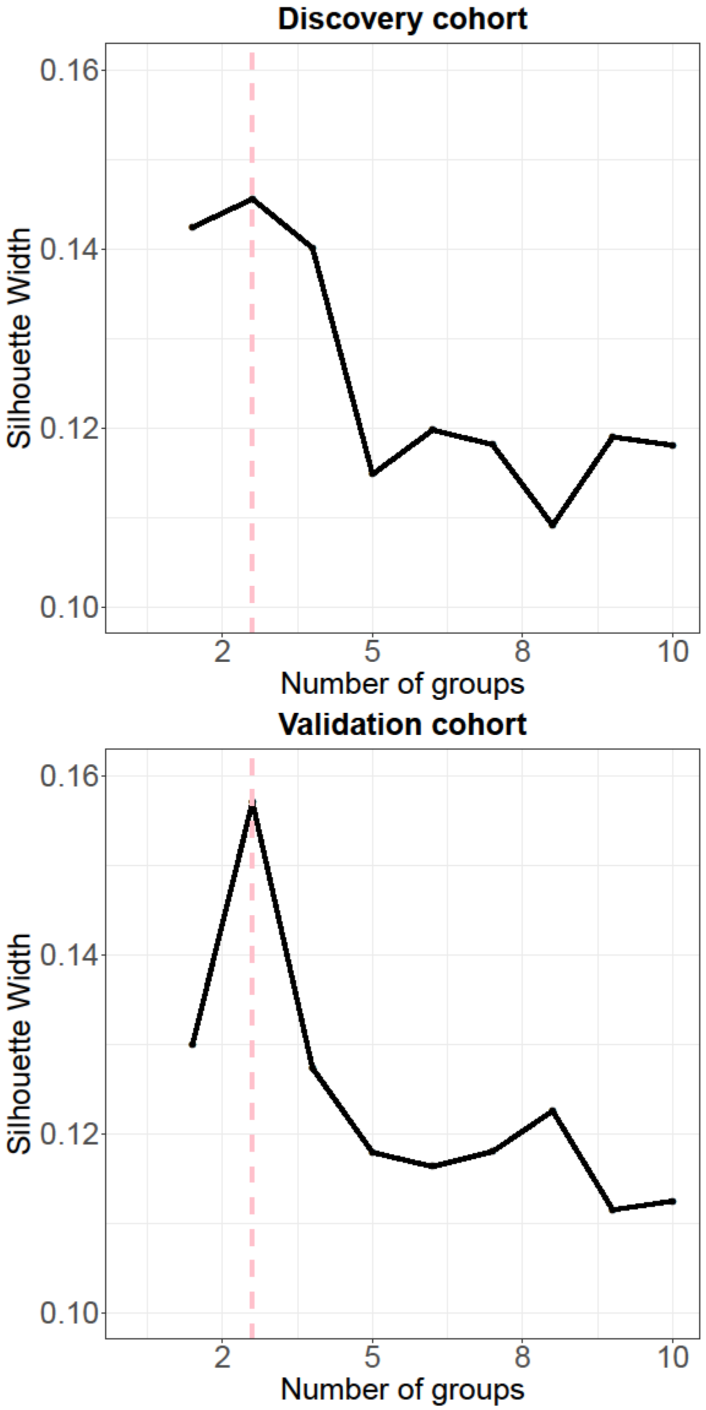

Supplementary Figure 2. Visualization the clustering results with t-distributed stochastic neighborhood embedding, or t-SNE method.

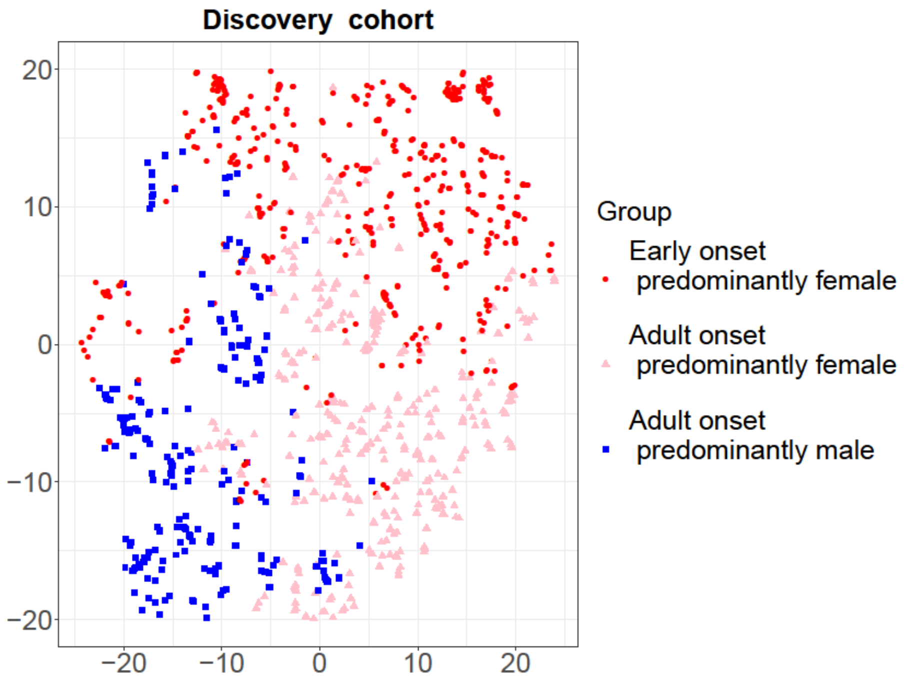

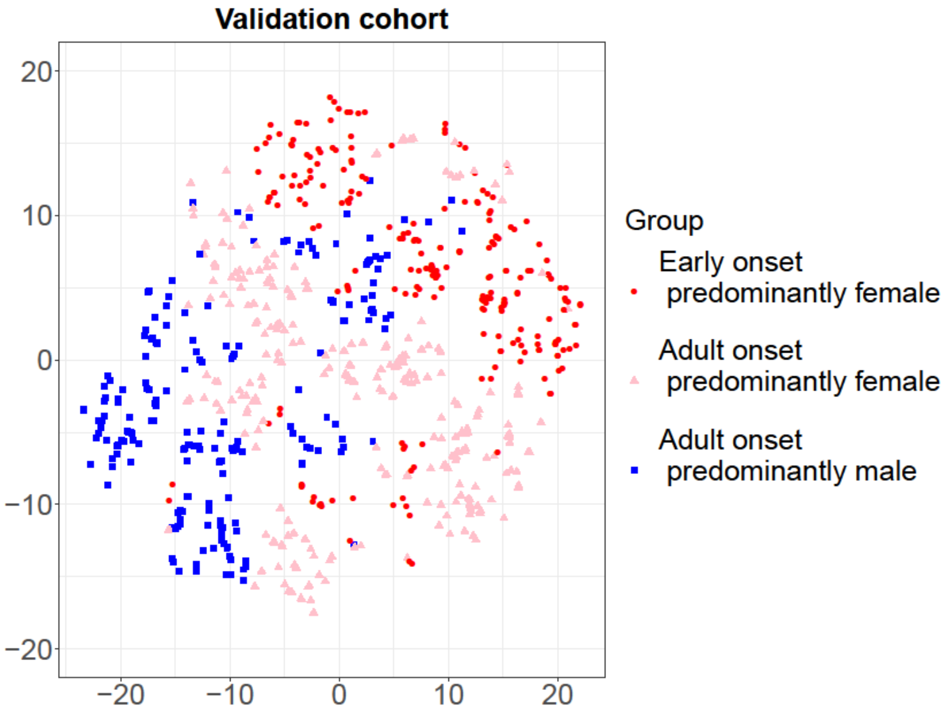

Supplement: Supplementary file 2 — Supplementary Info [file 41533_2020_168_MOESM2_ESM.pdf]
